# Nanotextured CeO_2_−SnO_2_ Composite: Efficient Photocatalytic, Antibacterial, and Energy Storage Fibers

**DOI:** 10.3390/nano13061001

**Published:** 2023-03-10

**Authors:** Jari S. Algethami, M. Shamshi Hassan, Touseef Amna, Faheem A. Sheikh, Mohsen A. M. Alhamami, Amal F. Seliem, M. Faisal, H. Y. Kim

**Affiliations:** 1 Department of Chemistry, College of Science and Arts, Najran University, Najran 11001, Saudi Arabia; 2 Promising Centre for Sensors and Electronic Devices (PCSED), Advanced Materials and Nano-Research Centre, Najran University, Najran 11001, Saudi Arabia; 3 Department of Chemistry, College of Science, Albaha University, Albaha 65799, Saudi Arabia; 4 Department of Biology, College of Science, Albaha University, Albaha 65799, Saudi Arabia; 5 Nanostructured and Biomimetic Lab, Department of Nanotechnology, University of Kashmir Hazratbal, Srinagar 190006, India; 6 Organic Materials and Fibers Engineering Department, Chonbuk National University, Jeonju 560011, Republic of Korea

**Keywords:** CeO_2_−SnO_2_, environmental remediation, antimicrobial, photocatalyst, supercapacitor

## Abstract

Bacterial infections remain a serious and pervasive threat to human health. Bacterial antibiotic resistance, in particular, lowers treatment efficacy and increases mortality. The development of nanomaterials has made it possible to address issues in the biomedical, energy storage, and environmental fields. This paper reports the successful synthesis of CeO_2_−SnO_2_ composite nanofibers via an electrospinning method using polyacrylonitrile polymer. Scanning and transmission electron microscopy assessments showed that the average diameter of CeO_2_−SnO_2_ nanofibers was 170 nm. The result of photocatalytic degradation for methylene blue dye displayed enhanced efficiency of the CeO_2_−SnO_2_ composite. The addition of SnO_2_ to CeO_2_ resulted in the enhancement of the light absorption property and enriched charge transmission of photoinduced electron–hole duos, which conspicuously contributed to momentous photoactivity augmentation. Composite nanofibers exhibited higher specific capacitance which may be accredited to the synergism between CeO_2_ and SnO_2_ particles in nanofibers. Furthermore, antibacterial activity was screened against *Escherichia coli* and CeO_2_−SnO_2_ composite nanofibers depicted excellent activity. The findings of this work point to new possibilities as an electrode material in energy storage systems and as a visible-light-active photocatalyst for the purification of chemical and biological contaminants, which would substantially benefit environmental remediation processes.

## 1. Introduction

Globally, environmental pollution is a serious problem, inflicting harm to life on the planet. Water pollution, among other types of pollution, has a significant negative impact on living species, including aquatic life. Water pollution is due to the discharge of harmful organic chemicals, such as dyes, acids, and antibiotics, into drinkable water bodies, such as rivers, lakes, and ponds, from textile, chemical, and pharmaceutical facilities. In nature, the majority of organic molecules are carcinogenic. Furthermore, water pollution sequentially causes soil pollution, which has a direct or indirect impact on daily living [1,2]. Synthetic color dyes, particularly those generated during textile washes, combine easily with water in comparison to chemicals and reagents; hence, the combination of effluents has hazardous potential. As a result, industrial effluent must be processed prior to being disposed of in the surroundings [3]. Dyes are colored aromatic organic complexes that capture light and provide color [4]. Because of these advantages, various dyes are utilized for a variety of applications in commerce, such as fabrics, foodstuffs, rubber, lithography, makeup, medication, plastic, concrete, and paper. Companies produce massive amounts of effluent, including carcinogenic and poisonous dyes, that pollute water and render it unsafe for human consumption. The textile sector is the largest dye-consuming business, relying on textile dyes, which are very complex molecules [5].

Methylene blue (MB), which is extensively used to color different fabrics, such as silk, wood, and cotton, as well as paper, is one of the most consumed ingredients in dye commerce [6]. Textile manufacturers typically release a considerable amount of MB dyes into natural water reservoirs, imposing health threats to beings and microbes [7]. Owing to its extreme toxicity, MB dye is dangerous to human health above a specific content level. MB is toxic, cancer-causing, and non-biodegradable, and it can put human well-being in danger and has a deleterious influence on the environment [7]. Human health dangers from MB comprise respiratory agony, gastrointestinal problems, vision, and digestive and mental illnesses [8]. Additionally, it produces indigestion, diarrhea, vomiting, cyanosis, dizziness, gastritis, jaundice, methemoglobinemia, tissue necrosis, and an escalation of heart rate, as well as untimely cell death in tissues and skin/eye irritability [7,9]. Contact with MB might cause skin redness and irritation [10]. For both aesthetic and toxicological reasons, MB liberation into surroundings poses a considerable concern. The management of wastewater containing MB dye before discharge into the environment is critical because of the negative effects on water quality and perception [11]. 

Likewise, major bacterial diseases are transmitted through wastewater. Therefore, microbiological management of drinking water should be the standard everywhere. Additionally, regular basic microbiological analysis of drinking water should be performed using culture methods to perceive the presence of pathogenic *Escherichia coli.* Hygienic water is essential for life, but an enormous number of persons are deprived of access to potable water, and many die as a result of waterborne bacterial infections [12]. To date, many water treatment technologies, including biodegradation, coagulation, adsorption, and photocatalysis, have been used to remove organic pollutants [13]. Multi-constituent photocatalysis of organic contaminants utilizing semiconducting NPs has gained popularity in recent decades because it is a low-cost, ecologically beneficial, and simple technology for treating dangerous contaminants in wastewater [4,14,15]. When compared with other ways, this technology is much more appealing due to the cheaper price of catalysts and the use of renewable energy [16].

One-dimensional (1D) nanostructured inorganic materials, especially nanofibers, are attracting a lot of attention. They were applied in various applications recently. One-dimensional metal oxides, as well as hybrids or composites [17], provide a wide range of material options, providing new options for future applications [18,19]. Electrospinning is one of the best flexible, multipurpose, and economical techniques that produce 1D nanofibers with a high aspect ratio, permeability, and adjustable penetrability. Remarkable progress was also made toward the utilization of such purposeful nanofibers in practical applications, such as fuel cells, lithium-ion arrays, solar booths, electronic sensors, photocatalysts, and supercapacitors [20]. Given the importance of renewable energy that is environmentally friendly, supercapacitors are in high demand; mostly because of their robust charge–discharge capacity, these supercapacitors are reliable [20].

Electrospinning has notable capability because of its proportionally low cost and relatively fast fabrication speed [21,22,23,24]. In recent years, cerium oxide (CeO_2_) has received significant attraction owing to its diverse properties, such as high-temperature stability and light absorption capability, as well as its broad range of catalytic applications [25,26,27]. It absorbs light in both the UV and visible regions; it was also applied as an effective oxidation catalyst, as it has high oxygen storage capability [28]. Moreover, it is also being used for the degradation of dyes and volatile organic compounds (VOCs) [29,30]. Currently, CeO_2_ is used as a photocatalyst and as an electrode material for supercapacitors [31,32]. Researchers are trying to enhance the properties of CeO_2_ for various applications by mixing or coupling with other semiconductors, such as CeO_2_/ZnFe_2_O_4_ [33], TiO_2_-WO_3_-CeO_2_ [34], CeO_2_/Ni-Al [35], CeO_2_/MnO_2_ [36], CeO_2_/SiO_2_ [37], CuO-CeO_2_ [38], CaO/CeO_2_ [39].

Tin oxide (SnO_2_) is an n-type semiconductor with a band gap of 3.6 eV. SnO_2_ has extensively been utilized as gas sensors, transparent conductive electrodes, solar cells, and photocatalysts [40,41,42]. It is expected that mixing SnO_2_ with CeO_2_ may enhance its activity for various applications. In the present study, CeO_2_−SnO_2_ composite nanofibers (NFs) were manufactured via electrospinning and screened for antibacterial, photocatalytic, and electrochemical applications. For decades, antibiotics have commonly been used to treat bacterial infections. However, the fast growth of antibiotic-resistant bacteria has caused several issues that place a significant burden on the medical community. As a result, the use of nanoparticles as an antibacterial substitute has been investigated. Regarding this situation, metal nanoparticles showed broad-spectrum antibacterial action. Furthermore, the use of nanomaterials in the biomedical area allows researchers to solve the issues of bacterial antibiotic resistance due to their distinct antibacterial mechanisms. Several metal and metal oxide-based nanomaterials were recently fully integrated into antibacterial applications and demonstrated remarkable performances [43]. Among them, Ce- and CeO_2_-based nanomaterials garnered a lot of attention across the world. Numerous investigations demonstrated that CeO_2_ nanoparticles have remarkable antibacterial activity [44,45]. Several investigations showed that CeO_2_ has an antibacterial impact on *Staphylococcus aureus* [46,47]. Furthermore, some research studies used agar diffusion and microdilution assays to investigate and confirm *P. aeruginosa*’s sensitivity to CeO_2_ [48]. In the same way, SnO_2_ also received attention as an antibacterial agent, where it was shown to inhibit the development of several bacterial strains, such as *S. aureus* and *E. coli* [49,50]. Furthermore, it was also found that SnO_2_ disinfects germs effectively once incapacitated with transition metal ions, for instance, Co@SnO_2_ and Ag@SnO_2_ nanoparticles exhibit powerful antimicrobial properties [51,52,53].

However, to the best of our knowledge, no research work has been published on the antibacterial behavior of CeO_2_−SnO_2_ composite NFs. This paper describes the facile manufacturing of CeO_2_−SnO_2_ composite NFs via electrospinning and their incredible photocatalytic, electrochemical applications, and antibacterial activity against *E. coli.* The composite was categorized using various physicochemical techniques, such as XRD, SEM, TEM, FT-IR, PL, and UV-vis. The photocatalytic activity was measured using MB disintegration under visible light radiation. The mechanism of improved photocatalytic activity of composite CeO_2_−SnO_2_ NFs was interpreted. The electrochemical efficiency of synthesized materials was scrutinized in terms of the cyclic voltammetry performance, which displayed that the CeO_2_−SnO_2_ composite possessed outstanding electrochemical efficacy as a supercapacitor material. Overall, the outcomes of this research highlight the innovative possibilities of utilizing these 1D high-aspect-ratio composite NFs as an electrode in energy storage systems and as a visible light active photocatalyst for the purification of chemical and biological contaminants, which would greatly assist in environmental remediation procedures. Henceforward, composites of these two materials (CeO_2_−SnO_2_) can serve as an excellent preliminary point for augmenting electrodes and photocatalytic efficiency. Additionally, this combination will be an imminent antimicrobial material with multiple functionalities. To recapitulate, the novelty of this work is that we used a facile electrospinning technique to fabricate composite NFs using cost-effective precursors. The CeO_2_−SnO_2_ composite NFs with a high aspect ratio are an auspicious future material and can be applied for antibacterial, photocatalyst, and electrode purposes, all in one.

## 2. Materials and Methods

### 2.1. Synthesis of Pure CeO_2_ and CeO_2_−SnO_2_ Composite NFs

Pristine CeO_2_ NFs were fabricated via an electrospinning process devoid of tin(II)ethylhexanoate in sol-gel. A polyvinylpyrrolidone (PVP, 15 wt%) solution was made via the usual technique by melting PVP in dimethylformamide (DMF) under magnetic stimulation for 5 h at room temperature. Ce(NO_3_)_3_·6H_2_O (1.0 g) was then added to the PVP solution while vigorously shaking. The resulting sol-gel was electrospun. The samples were subsequently sintered in air for 2 h at 500 °C to eliminate the polymer.

In the general process, for the synthesis of CeO_2_ and CeO_2_-SnO_2_ composite NFs, PVP (15 wt%) solution was primed by liquefying PVP in DMF on a magnetic stirrer as abovementioned. Tin(II) ethylhexanoate (0.15 mL) was supplemented with 2 mL of ethanol, then transferred into 10 mL of PVP solution. Ce(NO_3_)_3_·6H_2_O (1.0 g) was added to produce the final solution. The acquired sol-gel was transported into a 10 mL needle with a stainless steel spike. A copper pin coupled to a high voltage generator was implanted in solution as a positive terminal, while a ground iron barrel roofed with a polyethylene leaf assisted as the counter electrode. The mixture was retained as a capillary by regulating the inclination angle. A voltage of 17.5 kV was maintained to produce the final resultant assortment. The distance between the tip of the needle and the collector was held at 15 cm. Primarily, as-spun composite NFs were desiccated at 80 °C for 24 h in a vacuum. Additionally, to eliminate the polymer, composites were calcined in air at 500 °C at a rate of 2 °C/min for 2 h.

### 2.2. Classification of Pure CeO_2_ and CeO_2_−SnO_2_ NFs

The XRD outlines of pure and composite NFs were measured using a Rigaku/Max-3A (Tokyo, Japan) with Cu Kα radiation (λ = 1.540 Å) over Bragg angles between 20° to 80°. To scan surface features, pristine and composite NFs were examined using field emission scanning electron microscopy (FE-SEM, JSM6700, JEOL, Tokyo, Japan) and high-resolution transmission electron microscopy (HRTEM-H-7650 Hitachi, Co., Tokyo, Japan). The elemental conformation of the samples was scrutinized using energy-dispersive X-ray spectroscopy (EDS) coupled to an SEM instrument. The EDS of the sample was taken on carbon tape with a Pt coating. The photoluminescence (PL) spectra of the NFs were measured at room temperature using the 325 nm line of a He-Cd laser at a power of 25 mW for excitation (Kimmon Koha, JP/IK 3302 R). The light absorbance of NFs was measured by means of a UV-vis diffused reflectance spectrum (UV-DRS, 525 Shimadzu).

### 2.3. Photocatalytic Degradation

Photocatalytic activity of CeO_2_−SnO_2_ composite has been performed by disintegration of MB dye in visible light treatment using 450 W Xenon lamp. In 250 mL of a solution, there was a dye concentration of 10 mgL^−1^ and 250 mg of photocatalyst; this was stirred for 1 h in the dark (at a neutral pH). Afterward, around 3 mL of aliquots was taken at regular time intervals from the solution and centrifuged. The absorbance of MB dye mixture was analyzed by a UV–Vis spectrophotometer (Shimadzu UV-3101, UV Probe) at a 664 nm absorbance peak.

### 2.4. Electrochemical Characterization

Cyclic voltammetry was undertaken in a three-electrode configuration by means of a potentiostat (Digi-Ivy, USA). A glassy carbon electrode was used, while Ag/AgCl and Pt were used as the working standard and the counter electrode, respectively. The specific capacitance (Cs) was measured from CV curves calculated graphically by integrating the area under the CV curve with help of the following equation [54]:

(1) $$\mathrm{Cs}\;=\overset{\mathrm{Vc}}{\underset{\mathrm{Va}}{\int }}\mathrm{IVdV}\;\frac{1}{w\;\times \;\upsilon \;\times \left(\mathrm{Va}\;-\;\mathrm{Vc}\right)}$$

where w is the mass of the electrode and υ is the sweep rate (V/s).

### 2.5. Antimicrobial Activity

The antimicrobial actions of CeO_2_ and CeO_2_−SnO_2_ composite NFs were screened against *E. coli*, which is ever-present throughout the atmosphere. The bacterial standard cultures were held on nutrient agar (NA) plates. Bacterial culture from the agar plates was seeded in 5 mL NS solution and adjusted to 1 × 10^6^ CFU/mL. To evaluate the antibacterial effect, different concentrations of CeO_2_ NFs and CeO_2_−SnO_2_ composite NFs were prepared and screened. Preliminary screening was conducted using an agar diffusion technique, as described previously [55]. In short, 25 mL of agar holding 1 mL of microbial culture was placed in Petri dishes. Approximately 50 µL from each dosage was put into a 4 mm diameter well. Dishes were pre-incubated for 3 h at room temperature to enable the pre-diffusion of samples and then incubated for 24 h at 37 °C. As a negative control, DMSO was utilized, and the standard drug ciprofloxacin was used as the positive control. The inhibition potency was described as the absence of bacterial growth in the vicinity of holes and a caliper was used to determine the inhibition zone.

## 3. Results and Discussion

**Figure 1:**
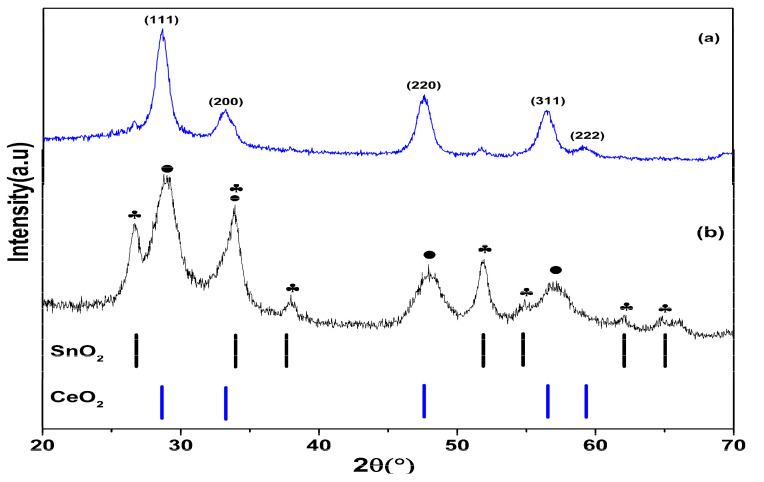
XRD patterns of (**a**) CeO_2_ and (**b**) CeO_2_−SnO_2_ composite NFs calcined at 500 °C.

Electrospinning is a simple and versatile technology that uses electrostatic repulsion between surface charges to extract nanofibers from a viscoelastic fluid and can be used for a variety of materials [56]. In the present study, it was utilized successfully to make composite NFs with diameters as small as hundreds of nanometers. The XRD configuration of CeO_2_ and CeO_2_−SnO_2_ NFs after calcination at 500 °C are displayed in Figure 1. Specifically, all of the reflection crests in Figure 1a were assigned to the cubic fluorite structure of CeO_2_ (JCPDS no. 65-2975) at angles of 28.5°, 33.2°, 47.5°, 56.4°, and 59.1°, which relate to the (111), (200), (220), (311), and (222) crystal planes, respectively [26]. A small peak was observed at 52.5°, which was probably the peak of Ce(OH)_3_ [57]. In the XRD configuration of CeO_2_−SnO_2_ (Figure 1b), major diffracted crests identical to those of CeO_2_ NFs were visualized. Additionally, diffraction peaks from the tetragonal SnO_2_ (JCPDS card no. 41-1445) were also observed [58], which demonstrates the effective materialization of CeO_2_−SnO_2_ composite NFs. No other mixes of Ce and Sn were detected using XRD.

**Figure 2:**
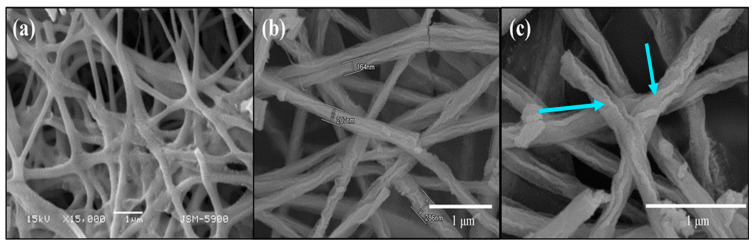
SEM profiles of (**a**) CeO_2_ and (**b**,**c**) CeO_2_−SnO_2_ nanofibers at low and high magnifications after calcination at 500 °C. The arrows clearly show the characteristic sheath on composite NFs, which was absent for the bare CeO_2_ NFs.

The morphologies of pure and composite NFs obtained after heating at 500 °C were demonstrated using SEM (Figure 2). From Figure 2a, it can be perceived that these arbitrarily oriented NFs had a uniform and continuous structure. Their lengths were measured as being several micrometers. The average diameter of the pure CeO_2_ NFs was 400 nm, whereas the average diameter of CeO_2_−SnO_2_ composite NFs was lower at 170 nm, which might have been due to the interaction between Ce and Sn (Figure 2b).

The arrows clearly show the characteristic sheath on the composite NFs, which was due to the presence of SnO_2_ (Figure 2c). The different salt content significantly increased the solution conductivity, resulting in thinner electrospun fibers. Due to increased spinnability in the presence of Ce and Sn precursors, uniform nanofibers were generated. Consequently, it was observed that a different salt addition had a considerable impact on the spinnability of the polymer solution. Additionally, because of the increased conductivity, the electric field accelerated the ions and the interactions between the polymer and the salt resulted in a less compact polymer structure. Our findings show that when salt (Ce and S) ions were propelled by an electric field, the polymer chain followed the ions’ motion, thereby decreasing the fiber diameter size. Analogous studies were reported for polymeric fibers by electrospinning in the presence of salts [59,60].

**Figure 3:**
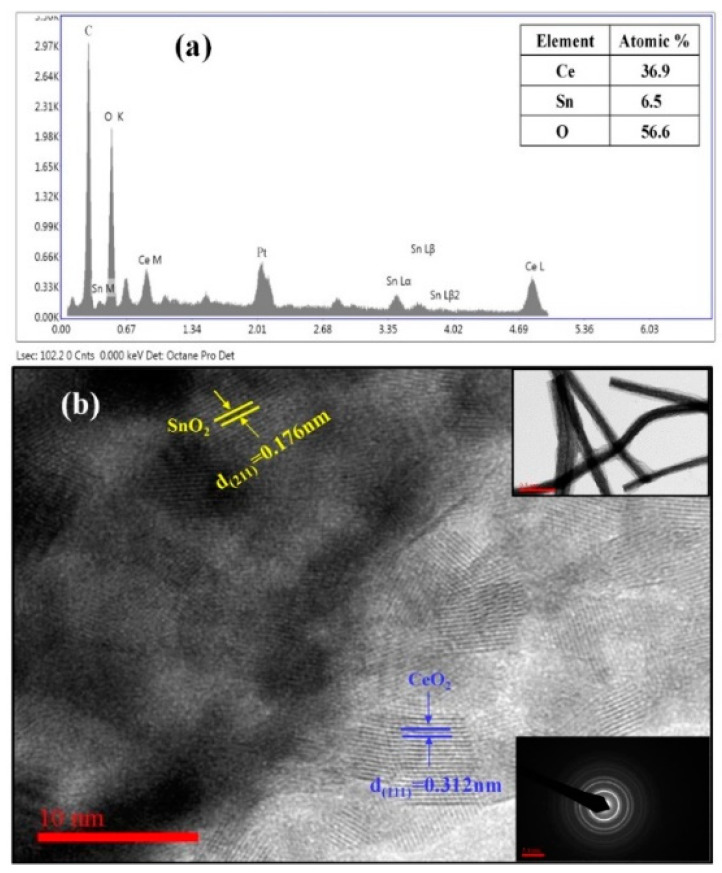
(**a**) EDX spectra and (**b**) HR-TEM image of the prepared CeO_2_−SnO_2_ composite NFs (the *inset* shows a low-resolution image and SAED pattern).

Figure 3a demonstrates the EDS results, which confirmed the presence of Ce, Sn, and O in the CeO_2_−SnO_2_ composite NFs. EDS analysis for the composite yielded an average atomic ratio of 1:0.15 for Ce/Sn. The detailed microstructure and morphology of composite NFs were further scanned using HR-TEM, the results of which are presented in Figure 3b. The surface consisted of two sets of lattices with spacings of 0.312 nm and 0.17 nm, corresponding to the interspace area between (111) and (211) of CeO_2_ and SnO_2_, respectively. It further verified the formation of a heterojunction between CeO_2_ and SnO_2_. The inset in Figure 3 demonstrates the low-resolution image of NFs. The TEM morphology of the CeO_2_−SnO_2_ NFs again showed that the average diameter of the composite NFs was 170 nm (*inset* of Figure 3). The corresponding selected area electron diffractions (SAED) arrangement of these NFs displayed a good ring pattern with no dislocations or imperfections, which can be attributed to the high crystalline phase of the composite sample, indicating the polycrystalline nature of the composite NFs (*inset* of Figure 3).

**Figure 4:**
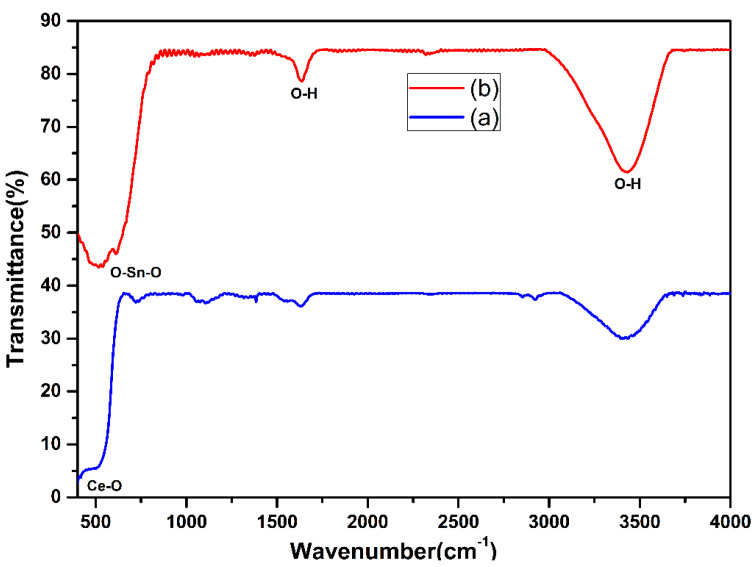
FT-IR configurations of the (**a**) CeO_2_ and (**b**) CeO_2_−SnO_2_ NFs.

Figure 4 displays the FT-IR bands of the virgin CeO_2_ and composite CeO_2_−SnO_2_ NFs. The existence of a strong band prior to 600 cm^−1^ could be assigned to the Ce-O-Ce stretching vibration [61] (Figure 4a). Both spectra revealed bands around 3450 cm^−1^ and 1637 cm^−1^ conforming to O–H widening vibration of the remaining water molecules and hydroxyl clusters. In the nanocomposite NFs spectra (Figure 4b), the occurrence of a band at 610 cm^−1^ corresponded to the stretching mode of SnO_2_ [62]. The presence of both CeO_2_ and SnO_2_ peaks suggested the integration of these materials in the final composite NFs.

**Figure 5:**
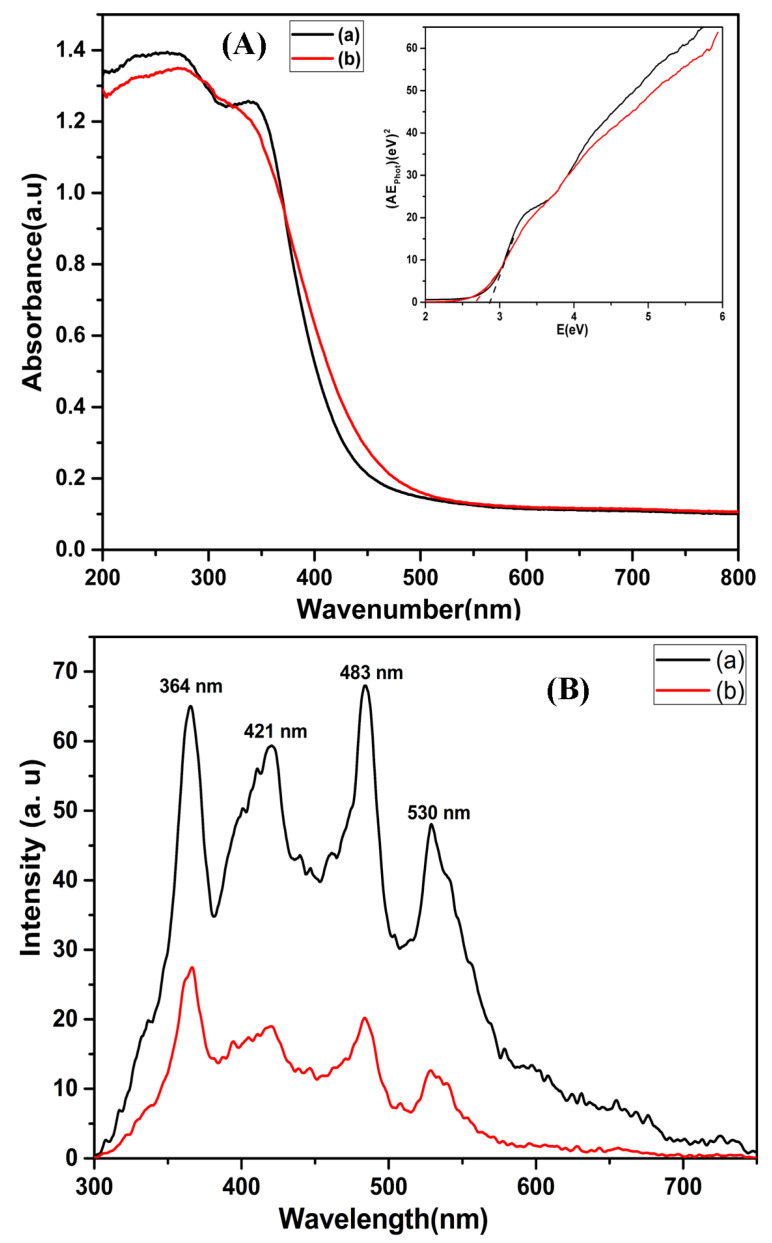
(**A**) UV-DRS pattern (*inset*: plot of the transformed Kubelka–Munk function versus the energy band gap) and (**B**) PL spectra of (**a**) CeO_2_ and (**b**) CeO_2_−SnO_2_ composite NFs.

The optical characteristics of bare and composite NFs were assessed using UV-DRS (Figure 5). Figure 5A demonstrates the UV-DRS results of the CeO_2_ and CeO_2_−SnO_2_ NFs, which show the absorption edge within the visible region. The spectrum of the CeO_2_-SnO_2_ composite presented a slight red shift, which coordinated well with pure CeO_2_ and could be ascribed to exciton partial leakage into the CeO_2_ matrix. To estimate the band gaps of the prepared samples, the modified Kubelka–Munk function was plotted for (Ahν)^2^ versus the energy of the exciting light (hν) using the following formula:

(2) $$\;\alpha =\frac{\mathrm{Constant}\;{\left(h\nu \;–\;E_{g}\right)}^{n}}{h\nu }$$

From the inset in Figure 5A, the calculated band gaps of the CeO_2_ NFs and CeO_2_−SnO_2_ composite were found to be 2.87 eV and 2.67 eV, respectively. This means that the smaller band gap of CeO_2_−SnO_2_ composite NFs will harness more visible light than CeO_2_ NFs. These results are consistent with an earlier report [63]. The PL emission spectra of the CeO_2_ and CeO_2_−SnO_2_ composite samples were documented using an excitation wavelength (Figure 5B). The PL spectrum of CeO_2_ had prominent peaks at 364, 421, 483, and 530 nm [64,65,66]. Moreover, the CeO_2_−SnO_2_ composite showed weaker emission peaks than CeO_2_, indicating a lower recombination frequency of the photogenerated electron–hole pair.

**Figure 6:**
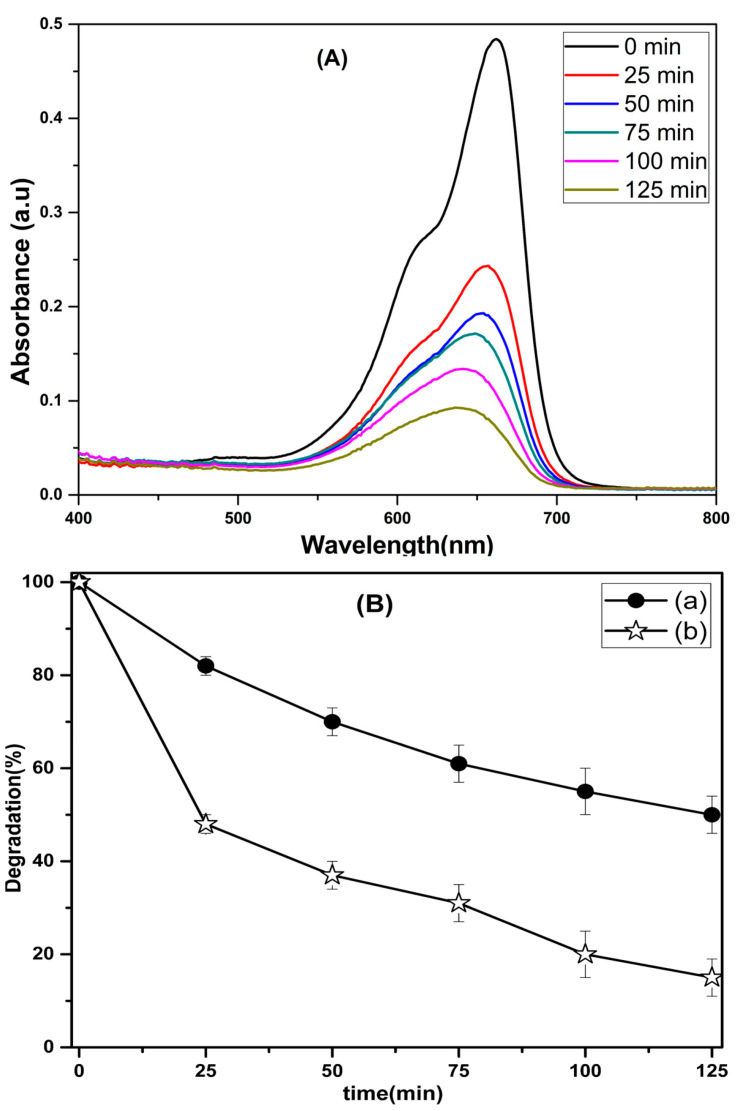
(**A**) Absorption spectra of MB dye using CeO_2_-SnO_2_ composite NFs for different periods and (**B**) relative photocatalytic action of (**a**) CeO_2_ and (**b**) a CeO_2_-SnO_2_ nanocomposite for MB dye.

The photoactivity of pure and composite nanofibers was assessed for the breakdown of MB dye under visible light (Figure 6). Figure 6A shows the MB concentration change calculated from its main absorption peak at 664 nm. From a comparative experiment, it can clearly be seen that CeO_2_−SnO_2_ composite photocatalyst displayed better photoactivity than the pure CeO_2_ NFs (Figure 6B). After 125 min of visible light irradiation, the photodegradation effectivenesses of the CeO_2_ and the CeO_2_−SnO_2_ composite photocatalysts for MB were 42% and 85%, respectively. The better photodegradation efficiency of the CeO_2_-SnO_2_ composite can be ascribed to the development of heterojunction between CeO_2_ and SnO_2_ and the parting of photogenerated electron–hole pairs.

**Figure 7:**
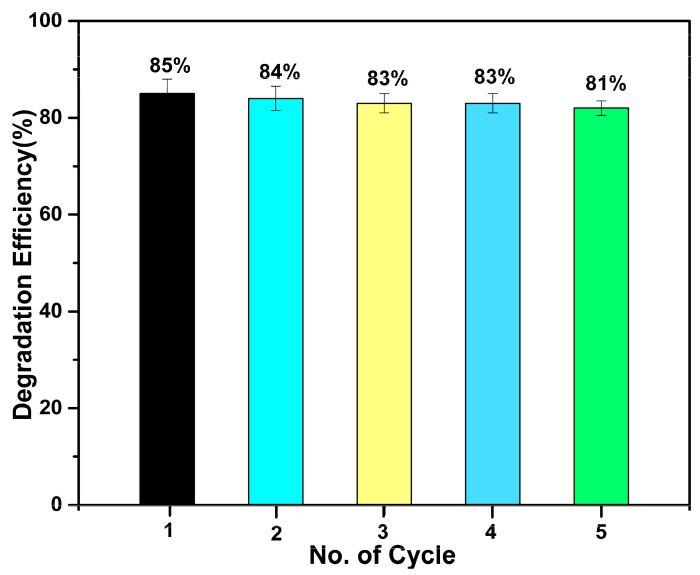
Recyclability studies of CeO_2_−SnO_2_ composite NFs for five consecutive cycles. Different color indicates different cycles

Figure 7 shows the reusability capacity of CeO_2_−SnO_2_ composite NFs for the degradation of MB dye. It was demonstrated that even after five cycles, the CeO_2_−SnO_2_ composite still exhibited good photocatalytic efficiency. It can be perceived that as the number of cycles increased, the composite photocatalytic activity marginally declined. The CeO_2_−SnO_2_ catalyst degraded 85% of the MB dye solution on its initial application, but after repeated applications (5th cycle), it had achieved a decolorization efficiency of 81%.

This shows that the CeO_2_−SnO_2_ catalyst was a reliable and efficient composite for dye degradation. Erstwhile, many similar studies also reported on composite nanomaterials for MB dye degradation (Table 1).

**Figure 8:**
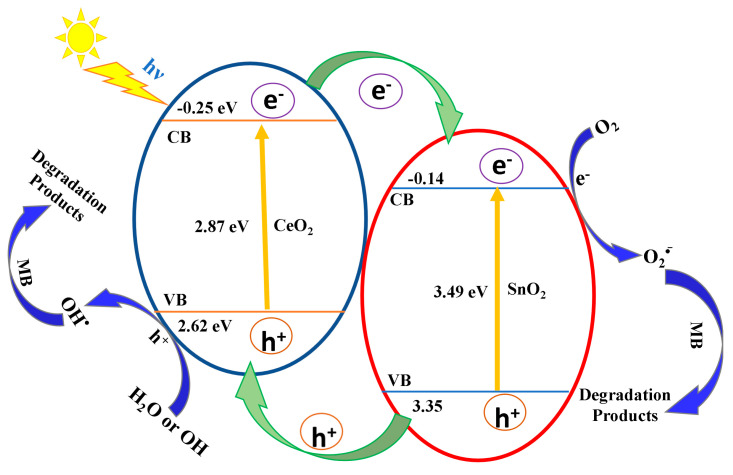
Schematic representation of the energy band configuration and electron−hole pair separation in CeO_2_−SnO_2_ NFs.

A potential mechanism for photocatalytic MB degradation using a CeO_2_−SnO_2_ composite is hypothesized and illustrated in Figure 8. Under visible light irradiation, photons are absorbed by the CeO_2_ catalyst when energy is equivalent to or greater than the band gap. CeO_2_ gets excited and generates electrons (e^−^) and holes (h^+^).

CeO_2_ + hν → (e_CB_^−^)_CeO2_ + (e_VB_^+^)_CeO2_ (3)

The photoexcited electrons are transported to the SnO_2_, as the conduction band (CB) position of CeO_2_ is more negative than SnO_2_. Meanwhile, the transported electrons in the CB of SnO_2_ will interact with dissolved oxygen molecules to generate superoxide radical anions (O_2_^−^). The superoxide radicals react with water and produce hydroperoxide radicals (HO_2_) and hydroxyl radicals (OH)**.** Subsequently, the holes on the valence band (VB) of SnO_2_ migrate to the VB of the CeO_2_ since the valence band potential of CeO_2_ is more negative than that of SnO_2_. Simultaneously, the generated h^+^ can oxidize water molecules to produce OH. These radicals have a strong ability to degrade dye molecules (Figure 8). The MB dye is photo-oxidized into carbon dioxide and water molecules or inorganic ions as degradation products [63].

O_2_ + (e_CB_^−^)_SnO2_ → O_2_^−^ (4)

O_2_^−^ + H_2_O → HO_2_ + OH^−^ (5)

h^+^ + H_2_O → OH ^−^ + H^+^ (6)

MB dye + Radical species (O_2_^−^, OH^−^) → Degradation products (CO_2_ + H_2_O) (7)

**Figure 9:**
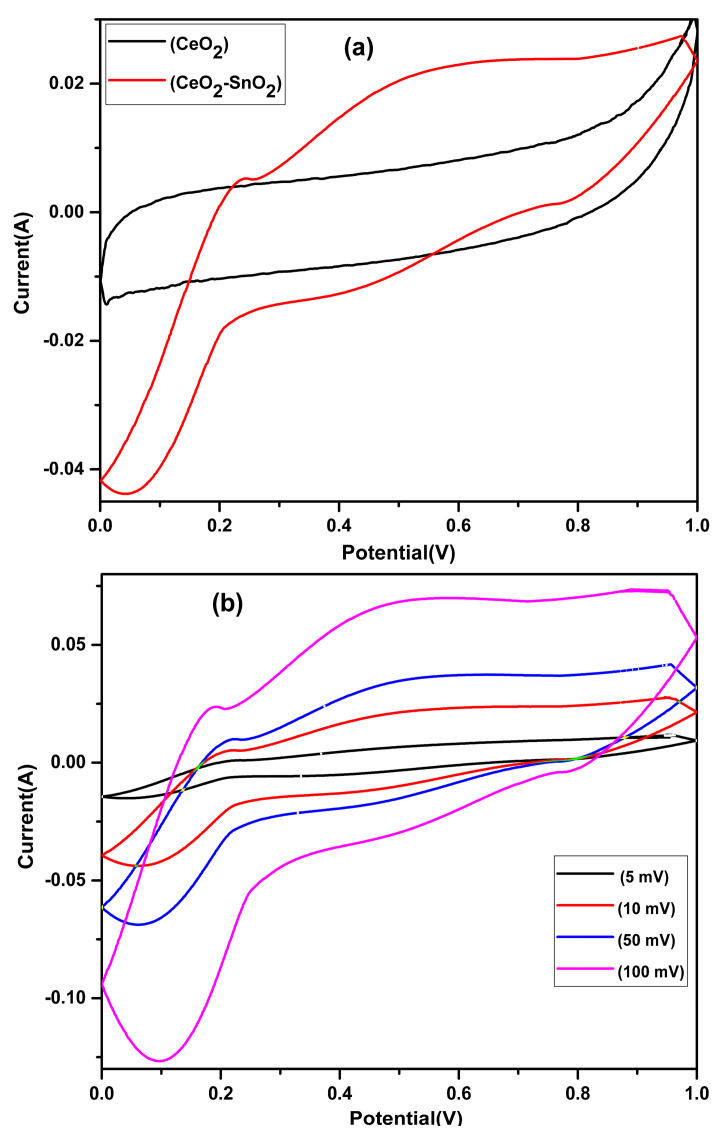
(**a**) Cyclic voltammograms of the CeO_2_ and CeO_2_−SnO_2_ NFs electrodes at a scanning rate of 5 mV/s and (**b**) CVs of CeO_2_−SnO_2_ NFs at different scanning rates.

Figure 9A demonstrates the cyclic voltammograms (CVs) of the pure CeO_2_ NFs and CeO_2_−SnO_2_ NFs. The CeO_2_−SnO_2_ composite NFs indicated higher current values and better voltammetric shapes compared with pure CeO_2_. The CeO_2_−SnO_2_ composite exhibited improved specific capacitance (413.9 Fg^−1^) compared with the CeO_2_ NFs (263.9 Fg^−1^) at a scan rate of 10 mV/s. No redox peaks were observed in the CV of the bare CeO_2_ NFs, which might have been due to the reason that there was little or no oxidation–reduction (corrosion or degradation) process going parallel to the double-layer charging. The presence of redox peaks in the CeO_2_−SnO_2_ composite NFs indicates that reversible and incessant faradaic oxidation–reduction responses were involved during the charging and discharging procedure [75,76]. It can be assumed that the electrochemical performances of composites were enhanced due to the synergistic effect between CeO_2_ and SnO_2_. Figure 9B shows the CV curves for different scan rates for the CeO_2_−SnO_2_ composite. The CeO_2_−SnO_2_ composite NFs confirmed elevated specific capacitance values of 413.9, 294.94, 147.6, and 16.1 F/g at scan rates of 5, 10, 50, and 100 mVs^−1^, correspondingly.

**Figure 10:**
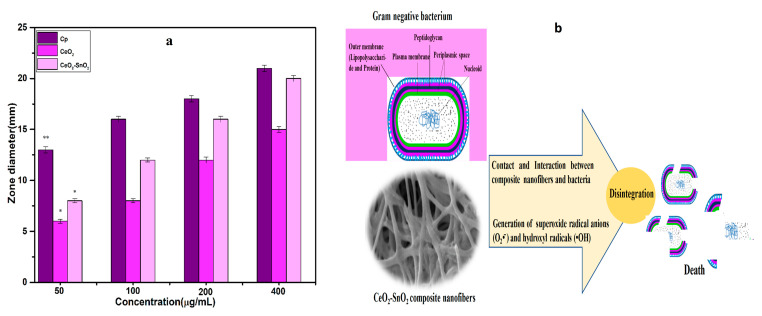
(**a**) *E. coli* susceptibility to CeO_2_ NFs and CeO_2_−SnO_2_ composite NFs (50–400 µg/mL). Ciprofloxacin was used as the standard antibiotic. The data are given as mean values and standard deviations of three replicates. * *p* < 0.005 and ** *p* < 0.01 vs. control. (**b**) Pictorial illustration of bacterial death and disintegration.

In the antibacterial bioassay, CeO_2_ NFs and CeO_2_−SnO_2_ composite NFs were tested against specified bacteria using an agar diffusion experiment at various doses. The CeO_2_ and CeO_2_−SnO_2_ composite NFs were found to be the most promising, with MICs of 50 µg/mL for *E. coli* (Figure 10). The augmented activity of the CeO_2_−SnO_2_ composite NFs might be attributed to their form and wide surface area [77], as well as the synergistic influence of CeO_2_ and SnO_2_ in the composite NFs. However, the antibacterial activities of the virgin CeO_2_ NFs and CeO_2_−SnO_2_ composite NFs were identical at a low concentration, the antibacterial impact varied with the increase in concentration and incubation time. The chemical components of the CeO_2_−SnO_2_ composite NFs primarily reacted with the bacterial outer cell wall before diffusing inside the inner wall, producing disarray and leaking through the disturbance in the inner cell content and distortion. A few previous investigations also showed that CeO_2_ nanoparticles have exceptional antibacterial activity [44,45]. In particular, some studies found that CeO_2_ has an antibacterial effect on *S. aureus* [46,47]. Furthermore, agar diffusion and microdilution experiments were utilized in certain studies to examine and validate *P. aeruginosa*’s sensitivity to CeO_2_ [48]. Similarly, SnO_2_ also attracted interest as an effective antibacterial, where it was shown to inhibit the development of several bacterial strains, such as *S. aureus* and *E. coli* [49,50]. Furthermore, it was observed that SnO_2_ supplemented with transition metal ions effectively disinfects microorganisms and possesses potent antibacterial activities [51,52,53].

## 4. Conclusions

In summary, CeO_2_−SnO_2_ composite NFs were prepared via electrospinning. As aforementioned, many strategies for removing MB and other textile dyes from industrial effluent were reported in the literature. However, in the present study, the photocatalysis approach was used. The addition of SnO_2_ to CeO_2_ significantly improved the photocatalytic MB degradation property and photoelectrochemical performance. After 125 min of irradiation, a photodegradation efficiency of 85% was achieved using the CeO_2_−SnO_2_ composite photocatalyst. Moreover, the CeO_2_−SnO_2_ composite NFs also showed higher specific capacitance. It is believed that these CeO_2_−SnO_2_ composite NFs with excellent photoactivity and electrochemical performance can be very promising for high-performance electrode materials in supercapacitors and as an efficient alternative photocatalyst. Rationally, due to their distinctive functional mechanism towards pathogens via reversible oxidation state transition between Ce(III) and Ce(IV), (IV) CeO_2_ nanoparticles with lower toxicity work as powerful antibacterial agents as well. The outcome of this study indicates the prospects of CeO_2_−SnO_2_ composite NFs to be exploited as an antimicrobial material, an electrode, and as a visible light active photocatalyst, which would greatly aid in the environmental cleanup process.

## Figures and Tables

**Table 1:** Comparison of photocatalytic work results of different composite nanomaterials for MB dye degradation.

No.	Photocatalyst	Dye	Light Source	Time (min)	Degradation Efficiency (%)	Reference
**1.**	V_2_O_5_/RGO composite	MB	UV/visible	100	98.85	[4]
**2.**	HAP−MnFe_2_O_4_ nanocomposites	MB	Visible	150	88	[43]
**3.**	CeO_2_−Cu_2_O composite nanofibers	MB	UV/visible	180	92	[67]
**4.**	Mo/N−doped TiO_2_ nanorods@CNFs	MB	Visible	180	79.8	[68]
**5.**	PANI nanotube@TiO_2_ composite	MB	Visible	300	85	[69]
**6.**	C−doped ZnO nanofiber	MB	Solar	30	>95	[70]
**7.**	TiO_2_/ZrO_2_ composite nanofibers	MB	Visible	180	82.7	[71]
**8.**	TiO_2_−decorated carbon nanofibers	MB	UV	180	97.4	[72]
**9.**	α−Fe_2_O_3_/Bi_2_MoO_6_ composite nanofibers	MB	Sunlight	240	94.8	[73]
**10.**	ZnO/CdO alloy nanofibers	MB	Visible	270	>90	[74]
**11.**	Nanotextured CeO_2_−SnO_2_ composite fibers	MB	Visible	125	85	This study

## Data Availability

Data is included in the main text.
